# Synthesis and Characterization of Novel [2 + 1] Tricarbonyl Rhenium Complexes with the Hydrophilic Phosphine Ligands PTA and CAP

**DOI:** 10.1155/2022/3117661

**Published:** 2022-06-13

**Authors:** Ioanna Roupa, Charalampos Flampouraris, Antonio Shegani, Myrto Ischyropoulou, Konstantina Makrypidi, Catherine Raptopoulou, Ioannis Pirmettis, Minas S. Papadopoulos, Vassilis Psycharis, Aristeidis Chiotellis

**Affiliations:** ^1^Institutes of Nuclear & Radiological Sciences & Technology, Energy & Safety, National Center for Scientific Research “Demokritos”, 15310 Athens, Greece; ^2^Institutes of Nanoscience and Nanotechnology, National Center for Scientific Research “Demokritos”, 15310 Athens, Greece

## Abstract

In the pursuit of hydrophilic model *fac*-[Re(CO)_3_]^+^ complexes for (radio) pharmaceutical applications, six novel [2 + 1] mixed-ligand complexes of the general type *fac*-[Re(CO)_3_(bid)P] were synthesized and characterized, where bid is a bidentate ligand bearing either (N, O) or (S, S′) donor atom sets and P is the hydrophilic phosphine 1,3,5-triaza-7-phosphoadamantane (PTA) or its macrocyclic homologue 1,4,7-triaza-9-phosphatricyclo[5.3.2.1]tridecane (CAP). The (N, O) ligands used in this study were picolinic and quinaldic acid, while the (S, S′) ligand was diethyldithiocarbamate. The complexes were synthesized in generally high yields and purity and the characterization was performed by spectroscopic methods, IR, NMR, and elemental analysis. Detailed X-ray crystallographic study of molecular packing by using Hirshfeld analysis tools revealed a plethora of intermolecular interactions such as hydrogen bond, *π*⋯*π*, C-H⋯*π*, and carbonyl-carbonyl interactions. To our knowledge, the CAP complexes reported herein are the first example of [2 + 1] mixed-ligand *fac*-[Re(CO)_3_]^+^ complexes with CAP. The new complexes might have the potential to serve as platforms for the design of target-specific complexes with favorable pharmacokinetics.

## 1. Introduction

Rhenium has recently attracted renewed attention in medicine due to its increasing potential applications in the anticancer arena. In fact, two isotopes of rhenium are *β*-emitters (^186^Re, *E*_max_ = 1.1 MeV, *t*_1/2_ = 90.6 h; ^188^Re, *E*_max_ = 2.1 MeV, *t*_1/2_ = 17 h) and therefore they are suitable candidates for therapeutic applications in radiotherapy [1]. In addition, rhenium shares a very similar chemistry with ^99m^Tc, the most widely used Single Photon Emission Computed Tomography (SPECT) radioisotope in Nuclear Medicine, and therefore any advancements in synthetic methodologies to access rhenium complexes can be usually applied for the development of more efficient ^99m^Tc radiopharmaceuticals [2]. Furthermore, recent research has shown that rhenium complexes possess potent anticancer properties while exhibiting diverse mechanisms of action, which makes them promising chemotherapeutic agents [3]. In view of the above, the exploration of rhenium's coordination chemistry remains an important aspect for the development of novel (radio) agents with optimal biological performance.

Increased hydrophilicity is often a central feature of pharmaceutical design because such compounds have favorable *in vivo* characteristics for medical applications [4] (e.g., increased aqueous media solubility and faster clearance of the drug from the body). The organometallic *fac*-[M(CO)_3_]^+^ core (M = ^nat^Re, ^186/188^Re and ^99m^Tc) is undoubtedly the most versatile precursor for the development of complexes of (radio) pharmaceutical interest. However, its increased lipophilicity can negatively influence the pharmacokinetic profile of the corresponding complexes [5]. This problem can be further exacerbated when common phosphines are used as ligands despite them being suitable for stabilizing metals in intermediate to low oxidation states as in the *fac*-[M(CO)_3_]^+^ core, thanks to their *π*-acceptor and *σ*-donor properties [6]. The high lipophilicity and molecular weight of common aryl-and alkyl phosphines usually render these ligands incompatible for target-specific (radio) pharmaceutical applications.

Nevertheless, the hydrophilicity of *fac*-[M(CO)_3_]^+^ compounds can be enhanced by utilizing polar pharmacological modifiers, i.e., biologically innocent polar moieties that lessen the overall lipophilicity of the (radio) pharmaceutical compunds [5, 7]. In this respect, hydrophilic ligands could exert the same role with minimal disruption of the complexes' structural identity. The phosphine 1,3,5-triaza-7-phosphoadamantane (PTA) and its recently synthesized higher homologue 1,4,7-triaza-9-phosphatricyclo[5.3.2.1]tridecane (CAP) [8] are excellent hydrophilic components of transition-metal compounds that are widely used in the fields of organometallic catalysis as well as for pharmaceuticals development [9, 10]. Both PTA and CAP are air-stable, resistant to oxidation, easy to synthesize, and especially PTA is highly water soluble. Upon P-coordination, the remaining three nitrogen atoms can participate in acid-base interactions in aqueous solutions, which can further impact their pharmacokinetic behavior (e.g., biodistribution and cell uptake by cancer cells) [11]. In addition, the nitrogen atoms are reactive under specific conditions, enabling its functionalization (e.g., N-alkylation) and thus, it could be used as a starting point for targeted drug design by tethering biologically active molecules. A further feature that makes CAP particularly interesting is its stereoelectronic properties. CAP combines strong electron-donating ability with an extremely reduced steric hindrance (cone angle = 109°) [12] making this phosphine ligand unique compared to more classical tertiary phosphines.

The coordination chemistry of PTA with transition metals has been extensively reported [9, 13, 14]. In relation to their use as medicinal compounds, ruthenium and platinum have attracted the main focus of relevant research since PTA complexes with these metals exhibit potent anticancer action [15–17]. However, the chemistry of PTA with rhenium is being increasingly investigated thanks to rhenium's promising medical and catalytical applications. PTA complexes have been reported with rhenium in almost all oxidation states ranging from (+VII) to (+I) [18–26]. Concerning *fac*-[Re^I^(CO)_3_]^+^ complexes, PTA is coordinated to the metal centre either in combination with other monodentate ligands (e.g., Cl and Br) [27] or as part of the [2 + 1] mixed-ligand approach where three labile aqua ligands on the *fac*-[M(CO)_3_(H_2_O)_3_]^+^ synthon are substituted by a bidentate ligand (bid) and PTA [28–32]. Still, the donor atom combinations with PTA in rhenium complexes incorporating the *fac*-[Re(CO)_3_]^+^ core remain limited, despite it being the most prominent synthon in radiopharmaceutical and related medicinal chemistry research. As for CAP, its' coordination chemistry with rhenium remains largely unexplored and only in combination with monodentate ligands [11].

In addition to the exploration of the [2 + 1] strategy for the design of new Re(I) complexes by using the appropriate ligands, there is an increased interest on the study of intermolecular interactions of Re(I) complexes in the solid state [33–35] based on diverse types of interactions of rhenium complexes with DNA [36, 37]. Similar systematic crystal structure studies [38–40] have revealed the importance of lone pair *π* and more specifically the role of carbonyl-carbonyl interactions on the supramolecular assembly of Re complexes. In an effort to develop a new platform of hydrophilic model *fac*-[Re(CO)_3_]^+^ complexes for radiopharmaceutical and/or medicinal chemistry applications, we report herein a series of novel [2 + 1] rhenium complexes of the general type *fac*-[Re(CO)_3_(bid)P], where P is PTA or CAP used as the polar, hydrophilic modifier and bid is either quinaldic and picolinic acid (representing N, O donor atom set) or diethyldithiocarbamate (representing S, S′ donor atom set). To our knowledge, this is also the first time where [2 + 1] mixed-ligand *fac*-[Re(CO)_3_(bid)(X)] complexes are reported with CAP. The syntheses of the corresponding complexes are presented along with their spectroscopic characterization by NMR, IR, and X-ray crystallography. Hirshfeld surface analysis tools were used to elucidate the intermolecular interactions of the synthesized complexes since the type of these interactions can impact the packing of the complexes and their pharmacokinetic behavior.

## 2. Materials and Methods

### 2.1. General Information

All reagents and starting materials were purchased from commercial suppliers and used without further purification. CAP [41], and [NEt_4_]_2_[ReBr_3_CO_3_] [42] were synthesized by following published procedures. All organic solvents were used as supplied (ACS or HPLC grade) unless otherwise noted. IR spectra were recorded on a Nicolet 6700 FT-IR (Thermo Scientific, USA) in the region 4000–500 cm^−1^. ^1^H and ^13^C-NMR spectra were obtained in DMSO-d_6_ at 25°C on a Bruker Avance DRX 500 or 250 MHz spectrometer. All ^31^P-NMR spectra were obtained on the 500 MHz spectrometer. The measured chemical shifts are reported in *δ* (ppm), and the residual signal of the solvent was used as the internal calibration standard (DMSO-d^6^: ^1^H = 2.50 ppm, ^13^C = 39.51 ppm). For the ^31^P-NMR, H_3_PO_4_ was used as internal reference. All ^13^C-NMR spectra were measured with complete proton decoupling. Data of NMR spectra were recorded as follows: *s* = singlet, *d* = doublet, *t* = triplet, *m* = multiplet, brs = broad singlet, br = broad signal). The coupling constant *J* is reported in hertz (Hz). The designations quin*H* and pic*H* denote the protons on the aromatic rings of quinaldic and picolinic acid, respectively. In analogy, the designations quin and pic demonstrate the carbons on the respective aromatic rings of the (N, O) ligands. Elemental analysis for C, H, and N was conducted on a PerkinElmer 2400 automatic elemental analyzer (PerkinElmer, USA). HPLC analysis was performed on a Waters 600 chromatography system (Waters, USA) coupled to a Waters 2487 Dual *λ* absorbance detector (Waters, USA). Separations were achieved on a Macherey-Nagel Nucleosil C-18 RP column (250 × 4 mm, 10 *μ*m) eluted with a binary gradient system including water/0.1% TFA (solvent A) and methanol/0.1% TFA as follows: 0-1 minute, 5% B; 1–11 minutes, 5–70% B; 11–26 minutes 70% B; 26-27 minutes 95% B; 27–42 minutes 95% B; 42–45 minutes 95–5% B. Flow rate, 1 ml/minute; UV detection, 254 and 220 nm.

### 2.2. Synthesis of the Rhenium Complexes

#### 2.2.1. fac-[Re(CO)_3_(quin) (H_2_O)], **1**

Complex **1** was synthesized according to a published procedure [43]. Briefly, [NEt_4_]_2_[ReBr_3_CO_3_] (231 mg, 0.30 mmol) and quinaldic acid (quin) (51.9 mg, 0.30 mmol) were dissolved in H_2_O (15 mL). The reaction mixture was stirred for 1 h at 60°C during which time a yellow precipitate formed. After cooling at room temperature, the yellow precipitate was filtered, washed with cold H_2_O, and dried under vacuum. NMR data are in agreement with those reported in the literature. Yield: 70% (97 mg). RP-HPLC: *t*_R_ = 17.0 minutes; IR (cm^−1^): 2027, 1903, 1877, 1644. Anal. Calc. for C_13_H_8_NO_6_Re: C: 33.91%, H: 1.75%, N: 3.04%. Found: C: 33.85%, H: 1.63%, N: 3.15%.

#### 2.2.2. fac-[Re(CO)_3_(quin) (PTA)], **1a**

Complex **1** (23.0 mg, 0.05 mmol) and PTA (8.0 mg, 0.05 mmol) were dissolved in MeOH (10 mL) and the yellow reaction mixture was refluxed for 2 h. The solvent was removed under reduced pressure to give **1a** after recrystallization from DCM/hexane. Yield: 94% (29 mg). RP-HPLC: *t*_R_ = 16.9 minutes. IR (cm^−1^): 2018, 1923, 1874, 1650. Anal. Calc. for C_19_H_18_N_4_O_5_PRe: C: 38.06%, H: 3.03%, N: 9.34%. Found: C: 38.23%, H: 3.15%, N: 9.28%. ^1^H-NMR (250 MHz, DMSO-d_6_, ppm): 8.93 (d, *J* = 7.4 Hz, 1H, quin*H*), 8.52 (d, *J* = 7.7 Hz, 1H, quin*H*), 8.41–8.23 (m, 2H, quin*H*), 8.23–8.08 (m, 1H, quin*H*), 8.04–7.83 (m, 1H, quin*H*), 4.29 (brs, 6H, NC*H*_*2*_N), 3.76 (brs, 6H, PC*H*_*2*_N). ^13^C-NMR (63 MHz, DMSO-d_6_, ppm): 194.59 (d, ^2^*J*_C-P_ = 8.1 Hz, *cis*-carbonyl), 194.44 (d, ^2^*J*_C-P_ = 8.1 Hz, *cis*-carbonyl), 189.62 (d, ^2^*J*_C-P_ = 70.1 Hz, *trans*-carbonyl), 172.10 (s, quin*C*OO), 152.1, 146.57, 142.07, 133.05, 130.54, 129.80, 129.66, 127.77, 122.98 (9 C *quin*COO), 71.38 (d, ^3^*J*_C-P_ = 7.2 Hz, N*C*H_2_N), 48.52 (d, ^1^*J*_P-C_ = 14.7 Hz, P*C*H_2_N). ^31^P-NMR (DMSO-d_6_, ppm): −71.65.

#### 2.2.3. fac-[Re(CO)_3_(quin) (CAP)], **1b**

Complex **1** (46.0 mg, 0.1 mmol) and CAP (21.9 mg, 0.11 mmol) were dissolved in MeOH (10 mL). The orange reaction mixture was refluxed for 2 h and evaporated to dryness under vacuum. Recrystallization from DCM/hexane afforded the product as an orange crystalline solid. Yield: 52% (35.0 mg). RP-HPLC: *t*_R_ = 17.5 minutes; IR (cm^−1^): 2019, 1926, 1877, 1646 cm^−1^. Anal. Calc. for C_22_H_24_N_4_O_5_PRe: C: 41.18%, H: 3.77%, N: 8.73%. Found: C: 41.06%, H: 3.69%, N: 8.82%. ^1^H-NMR (500 MHz, DMSO-d_6_, ppm): 8.91 (d, *J* = 8.4 Hz, 1H, quin*H*), 8.60 (d, *J* = 8.80 Hz, 1H, quin*H*), 8.32 (d, *J* = 8.4 Hz, 1H, quin*H*), 8.28–8.24 (m, 1H, quin*H*), 8.21–8.16 (m, 1H, quin*H*), 7.98–7.92 (m, 1H, quin*H*), 3.15–2.99 (m, 6H, PCH_2_N), 2.96–2.80 (m, 6H, N(CH_2_)_2_N), 2.71–2.59 (m, 6H, N(CH_2_)_2_N). ^13^C-NMR (125.76 MHz, DMSO-d_6_, ppm): 195.76 (d, ^2^*J*_C-P_ = 5.7 Hz, 2 x *cis*-carbonyls), 190.94 (d, ^2^*J*_C-P_ = 58.3 Hz, *trans*-carbonyl), 172.06 (quin*C*OO), 152.21, 146.57, 141.84, 133.02, 130.30, 129.70, 129.64, 127.91, 122.79 (9C, *quin*COO), 50.88 (N(*C*H_2_CH_2_)N), 50.77 (N(CH_2_*C*H_2_)N), 48.32 (d, ^1^*J*_P-C_ = 8.5 Hz, P*C*H_2_N), ^31^P-NMR (DMSO-d_6_, ppm): 38.66.

#### 2.2.4. fac-[Re(CO)_3_(pic) (H2O)], **2**

Complex **2** was synthesized according to a published procedure [44]. Briefly, [NEt_4_]_2_[ReBr_3_CO_3_] (231 mg, 0.30 mmol) and picolinic acid (pic) (74 mg, 0.60 mmol) were dissolved in Η_2_Ο (15 mL) and the reaction mixture was stirred for 3 h at 70°C. After cooling to room temperature, the volume of the solvent was reduced to ∼3 mL and was placed in the fridge overnight. The yellow precipitate that formed was filtered, washed with cold H_2_O, and dried under vacuum. NMR data are in agreement with that reported in the literature. Yield: 68% (84 mg). RP-HPLC: *t*_R_ = 14.9 min; IR (cm^−1^): 2024, 1895, 1874, 164; Anal. Calc. for C_9_H_6_NO_6_Re: C: 26.34%, H: 1.47%, N: 3.41%. Found: C: 26.26%, H: 1.36%, N: 3.38%.

#### 2.2.5. fac-[Re(CO)_3_(pic) (PTA)], **2a**

Complex **2** (30 mg, 0.07 mmol) and PTA (10 mg, 0.07 mmol) were dissolved in MeOH (7 mL) and the yellowish reaction mixture was refluxed for 2 h. The solvent was then removed under reduced pressure and the residue was recrystallized from DCM/hexane to afford **2a**. Yield: 76% (32.0 mg). RP-HPLC: *t*_R_ = 15.2 minutes. IR (cm^−1^): 2022, 1946, 1878, 1650. Anal. Calc. for C_15_H_16_N_4_O_5_PRe: C: 32.79%, H: 2.94%, N: 10.20%. Found: C: 32.65%, H: 3.02%, N: 10.16%. ^1^H-NMR (500 MHz, DMSO-d_6_, ppm): 8.81 (brs, 1H, pic*H*), 8.34–8.25 (m, 1H, pic*H*), 8.11 (d, *J* = 7.2 Hz, 1H, pic*H*), 7.89–7.79 (m, 1H, pic*H*), 4.42–4.28 (m, 6H, NC*H*_2_N), 3.84 (brs, 6H, PC*H*_2_N). ^13^C-NMR (125.76 MHz, ppm): 194.78 (d, ^2^*J*_C-P_ = 7.6 Hz, *cis*-carbonyl), 193.94 (d, ^2^*J*_C-P_ = 7.6 Hz, *cis*-carbonyl), 190.03 (d, ^2^*J*_C-P_ = 70.8 Hz, *trans*-carbonyl), 170.87 (pic*C*OO), 153.29, 140.80, 129.29, 126.94, 71.49 (d, ^3^*J*_P-C_ = 6.7 Hz, N*C*H_2_N), 48.19 (d, ^1^*J*_P-C_ = 14.4 Hz, P*C*H_2_N). ^31^P-NMR (DMSO-d_6_, ppm): −73.26.

#### 2.2.6. fac-[Re(CO)_3_(pic) (CAP)], **2b**

Complex **2** (41.0 mg, 0.10 mmol) and CAP (21.9 mg, 0.11 mmol) were dissolved in MeOH (10 mL). The yellow reaction mixture was refluxed for 2 h and the solvent was removed under reduced pressure to give **2b** after recrystallization from DCM/hexane. Yield: 97% (59.0 mg). RP-HPLC: *t*_R_ = 15.9 minutes. IR (cm^−1^): 2010, 1891, 1869, 1660. Anal. Calc. for C_18_H_22_N_4_O_5_PRe: C: 36.55%, H: 3.75%, N: 9.47%. Found: C: 36.47%, H: 3.62%, N: 9.53%. ^1^H-NMR (500 MHz, DMSO-d_6_, ppm): *δ* = 8.90 (brs, 1H, pic*H*), 8.31–8.21 (m, 1H, pic*H*), 8.13–8.05 (m, 1H, pic*H*), 7.86–7.77 (m, 1H, pic*H*), 3.22–3.11 (m, 6H, PCH_2_N), 3.00–2.89 (m, 6H, N(CH_2_)_2_N), 2.79–2.66 (br, 6H, N(CH_2_)_2_N). ^13^C-NMR (125.76 MHz, DMSO-d_6_, ppm): 196.13 (d, ^2^*J*_C-P_ = 5.1 Hz, *cis*-carbonyl), 195.23 (d, ^2^*J*_C-P_ = 5.1 Hz, *cis*-carbonyl), 191.38 (d, ^2^*J*_C-P_ = 59.1 Hz, *trans*-carbonyl), 170.95 (picCOO), 153.57, 149.63, 140.62, 129.11, 126.76 (5C, *pic*COO), 51.03 (N(*C*H_2_CH_2_)N), 50.74 (N(CH_2_*C*H_2_)N), 47.94 (d, ^1^*J*_C-P_ = 8.8 Hz, P(CH_2_)N). ^31^P-NMR (DMSO-d_6_, ppm): 37.21.

#### 2.2.7. fac-[Re(CO)_3_(Et_2_SS) (PTA)], **3a**

[NEt_4_]_2_[ReBr_3_CO_3_] (77.0 mg, 0.10 mmol), PTA (15.7 mg, 0.10 mmol), and sodium diethyldithiocarbamate trihydrate (Et_2_SS) (22.5 mg, 0.10 mmol) were dissolved in MeOH (4 mL). The reaction mixture was refluxed for 2 h and after cooling to room temperature, the solid that formed was filtered off, washed with cold MeOH, and dried under vacuum. Yield: 42% (25.0 mg). RP-HPLC: *t*_R_ = 18.8 minutes. IR (cm^−1^): 2008, 1914, 1878. Anal. Calc. for C_14_H_22_N_4_O_3_PReS_2_: C: 29.21%, H: 3.85%, N: 9.73%. Found: C: 29.18%, H: 3.72%, N: 9.85%. ^1^H-NMR (250 MHz, ppm): 4.46 (s, 6H, NCH_2_N), 4.18 (s, 6H, PCH_2_N), 3.63 (m, 4H, NCH_2_CH_3_), 1.23 (t, ^3^*J* = 7.1 Hz, 6H, NCH_2_CH_3_). ^13^C-NMR (63 MHz, DMSO-d_6_, ppm): *δ* = 209.37 (d, ^3^*J*_C-P_ = 2 Hz, S=C(S)N), 191.83 (d, ^2^*J*_C-P_ = 8.2 Hz, 2 x *cis*-carbonyls), 190.26 (d, ^2^*J*_C-P_ = 69.9 Hz, *trans*-carbonyl), 71.91 (d, ^3^*J*_P-C_ = 6.5 Hz, NCH_2_N), 49.10 (d, ^1^*J*_P-C_ = 16.3 Hz, PCH_2_N), 44.09 (NCH_2_CH_3_), 12.11 (NCH_2_CH_3_). ^31^P-NMR (DMSO-d_6_, ppm): −84.56.

#### 2.2.8. fac-[Re(CO)_3_(Et_2_SS) (CAP)], **3b**

[NEt_4_]_2_[ReBr_3_CO_3_] (77.0 mg, 0.10 mmol), CAP (21.9 mg, 0.11 mmol) and Et_2_SS (22.5 mg, 0.10 mmol) were dissolved in MeOH (4 mL). The reaction mixture was refluxed for 2 h at which time a yellow precipitate formed. The solid was filtered, washed with cold MeOH, and dried under vacuum. Yield: 45% (29 mg). RP-HPLC: *t*_R_ = 19.9 minutes. IR (cm^−1^): 1998, 1898, 1858. Anal. Calc. for C_17_H_28_N_4_O_3_PReS_2_: C: 33.05%, H: 4.57%, N: 9.07%. Found: C: 33.16%, H: 4.49%, N: 9.16%. ^1^H-NMR (250 MHz, DMSO-d_6_, ppm): *δ* = 3.74–3.47 (m, 10H, NC*H*_*2*_CH_3_ overlapping with PCH_2_N), 3.14–2.96 (m, 6H, N(CH_2_)_2_N), 2.96–2.75 (m, 6H, N(CH_2_)_2_N), 1.20 (t, ^3^*J* = 7.1 Hz, 6H, NCH_2_CH_3_), ^13^C-NMR (63 MHz, DMSO-d_6_, ppm): *δ* = 209.06 (s, SSCN), 193.16 (d, ^2^*J*_C-P_ = 6.7 Hz, 2 x *cis*-carbonyls), 191.23 (d, ^2^*J*_C-P_ = 57.4 Hz, *trans*-carbonyl), 51.06 (s, N(CH_2_)_2_N), 48.88 (d, ^1^*J*_P-C_ = 10.6 Hz, PCH_2_N), 43.94 (s, N*C*H_2_CH_3_), 12.25 (s, NCH_2_CH_3_). ^31^P-NMR (DMSO-d_6_, ppm): 29.17.

### 2.3. X-ray Crystal Structure Determination

A crystal of **1b** (0.10 × 0.20 × 0.40 mm) and a crystal of **3a** (0.08 × 0.18 × 0.41 mm) were mounted in air. A crystal of **2b** (0.08 × 0.22 × 0.53 mm) and a crystal of **3b** (0.05 × 0.21 × 0.26 mm) were taken from the mother liquor and immediately cooled to −113°C. Diffraction measurements were made on a Rigaku *R*-AXIS SPIDER Image Plate diffractometer using graphite monochromated Mo K*α* radiation. Data collection (*ω*-scans) and processing (cell refinement, data reduction, and empirical absorption correction) were performed using the CrystalClear program package [45]. Important crystallographic data are listed in Table 1. The structures were solved by direct methods using SHELXS v.2013/1 and refined by full-matrix least-squares techniques on *F*^2^ with SHELXL ver.2014/6 [46, 47]. All hydrogen atoms were located either by difference maps and were refined isotropically or were introduced at the calculated positions as riding on bonded atoms. All non-hydrogen atoms were refined anisotropically. Plots of the structure were drawn using the Diamond 3 program package [48]. The *CrystalExplorer* package V.17.5 [49] was used for the Hirshfeld Surface (HS) analysis studies of **1b**, **2b**, **3a,** and **3b**. For the HS studies, the dnorm and shape decorated surfaces were used together with the fingerprint plots. *d*_norm_ is a normalized contact distance, defined in terms of *d*_e_, *d*_i_ and the Van der Waals (VdW) radii of two atoms at a distance *d*_e_ outside from a point on the surface and at a distance *d*_i_ inside the surface correspondingly [50].

## 3. Results and Discussion

### 3.1. Synthesis of the Rhenium Complexes

**Scheme 1:**
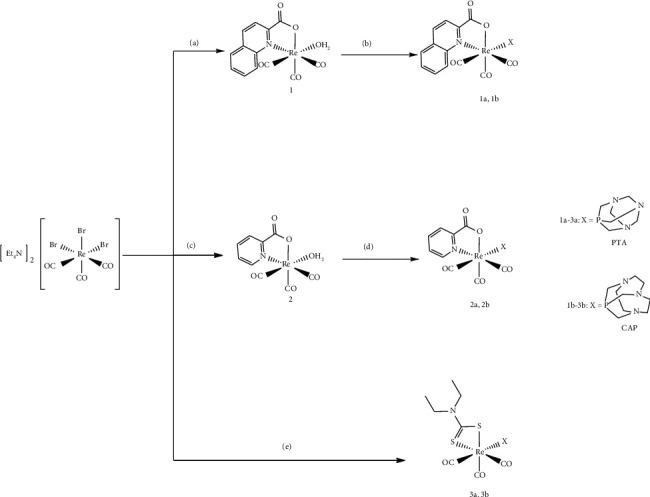
Synthesis of complexes **1a-3a** and **1b-3b**. Reactions and conditions: (a) quinH, H_2_O, 60°C, 1 h; (b) X, MeOH, reflux, 2 h; (c) picH, H_2_O, 70°C, 3 h; (d) X, MeOH, reflux, 2 h; (e) Et_2_SS, X MeOH, reflux, 2 h.

The synthesis of the new complexes is depicted in Scheme 1 [NEt_4_]_2_[Re(CO)_3_Br_3_] was used as the rhenium starting synthon for all syntheses. Complexes **1a-1b** and **2a-2b** were synthesized via the mono-aqua complexes **1** and **2,** respectively, formed by the reaction of [NEt_4_]_2_[Re(CO)_3_Br_3_] with the corresponding (N, O) ligand in water. The aqua Re complexes were then reacted with equimolar amounts of PTA or CAP in refluxing methanol to afford the final [2 + 1] complexes. However, complexes **3a-3b** were synthesized more efficiently by a one-pot reaction in methanol, using equimolar amounts of the rhenium precursor, diethyldithiocarbamate, and the corresponding phosphine. Pure compounds were obtained in moderate to excellent yields (42% to 97%) and were characterized by elemental analysis, IR, and NMR-spectroscopy. Crystals suitable for X-ray crystallography were obtained by slow evaporation from DCM/hexane.

### 3.2. IR Characterization

The IR spectra of all rhenium complexes show the typical pattern for the tricarbonyl *fac*-[Re(CO)_3_]^+^ moiety with bands in the range of 2013–1870 cm^−1^ [51, 52]. The presence of the strong band at ∼1604–1642 cm^−1^ is attributed to the stretching of the carboxylate carbonyl shifted to a lower frequency compared to that of free quinaldic acid at 1695 cm^−1^ [53] and picolinic acid at 1720 cm^−1^.

### 3.3. NMR Characterization

The ^1^H, ^13^C, and ^31^P-NMR data obtained for the synthesized complexes are consistent with the proposed structures. Upon coordination of the bidentate and PTA/CAP ligands, characteristic shifts are prominent compared to its non-coordinated states. In the (N, O) complexes, downfield shifts of all aromatic protons of the picolinic and quinaldic acid moiety are noted, attributed to the loss of electron density after coordination of the N-aromatic nitrogen. Coordination of the carboxylate moiety is confirmed by the downfield shift of its carbonyl peak (e.g., 172.10 ppm for complex **1a** compared to the corresponding peak of free quinaldic acid at 167.25 ppm).

The ^1^H-NMR peaks are generally broad indicating a degree of conformational mobility in the compounds and as such, no ^1^H-^31^P couplings can be seen for the coordinated phosphine ligands. Nevertheless, ^13^C-^31^P couplings are present both in the carbonyl peaks and the PTA/CAP carbons of the new complexes. The carbonyl at the *trans*-position to the phosphine ligands couples strongly with the P atom displaying a large coupling constant (e.g., ^2^*J*_C-P_ = 70.1 Hz at 194.44 ppm for **1a**) while a much smaller one is observed for the carbonyls at *cis*-position (e.g., ^2^*J*_C-P_ = 8.1 Hz at 194.59 ppm for **1a**) (Table 2), a fact that has been previously reported by us in similar systems [54] and others [55]. Interestingly, this ^2^*J*_C-P_ coupling is stronger for the PTA complexes compared to the corresponding CAP complexes (e.g., ^2^*J*_C-P (trans)_ = 58.3 Hz and ^2^*J*_C-P (cis)_ = 5.7 Hz for **1b**). In all PTA complexes, the carbons of the phosphine ligand display the expected ^13^C-^31^P couplings (^1^*J*_C-P_ for PCH_2_N and ^3^*J*_C-P_ for NCH_2_N) and appear as doublets. For the CAP complexes, ^13^C-^31^P couplings are observed only for the P*C*H_2_N carbon, while the N*C*H_2_*C*H_2_N carbons appear as two differentiated singlets in close proximity (for the N, O complexes) or as one singlet (for the S, S complex). This difference between the (N, O), and (S, S) complexes with CAP could be attributed to the larger degree of asymmetry due to the bulkier (N, O) ligand.

Finally, the ^31^P-NMR data of all PTA complexes (**1a-3a**) display significant downfield shifts compared to the free phosphine ligand due to loss of electron density from the P atom, providing proof of coordination of the phosphorus ligand to the *fac*-[Re(CO)_3_]^+^ metal centre (Table 2). The large difference between the ^31^P-NMR chemical shifts of uncoordinated PTA (−104.01 ppm) and CAP (+47.08 ppm) ligands suggest that these sterically similar phosphanes have substantially different electronic structures [10, 56]. This abnormal behavior is also reflected to the CAP derivatives **1b-3b**. As shown in Table 2, their ^31^P resonances are displaced upfield which is unusual among phosphane ligands but is also observed for other CAP complexes [12]. All complexes reported herein show a single ^31^P peak demonstrating the absence of isomers, as expected.

### 3.4. Description of the Structures

**Figure 1:**
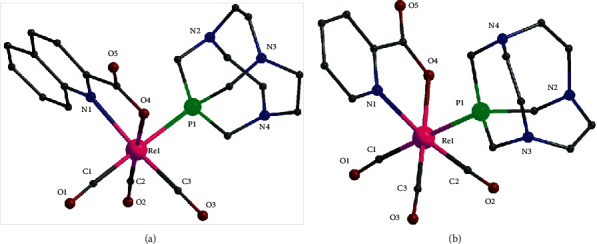
Partially labelled plots of **1b** (a) and **2b** (b).

The molecular structures of **1b** and **2b** are shown in Figure 1; selected bond distances and angles are listed in Table 3. Both complexes consist of the *fac*-[Re^I^(CO)_3_] moiety bound to one bidentate (N, O) chelate ligand and to the phosphorus atom of one CAP ligand. The (N, O) chelate ligand in **1b** is the anion of quinaldic acid and in **2b** is the anion of picolinic acid. The coordination geometry around the Re^I^ ion is distorted octahedral in both **1b** and **2b**. There are two short Re-CO bond distances (∼1.90 Å) in **1b** which are in the *trans*-position with respect to the (N, O) donor atoms of quinaldato ligand and one longer Re-CO distance at ∼1.94 Å which is in the *trans*-position to the Re-P bond. The Re-(N, O) bond distances are ∼2.24 and ∼2.14 Å, respectively. The Re-P bond distance is the longest in the coordination sphere (∼2.45 Å). The *cis*-angles in the coordination sphere are in the range ∼75–105° and the trans-angles are 170.84(12), 175.59(9), and 179.34(12)°. The five-membered ring in the coordination sphere, defined by Re-N-C-C-O, is almost planar with the largest deviation ∼0.11 Å for C(4). In **2b**, there are two long Re-CO bond distances (∼1.94 Å), *trans* to the (N, O) donor atoms of picolinato ligand, and one shorter Re-CO distance at ∼1.88 Å which is *trans* to the Re-P bond. The Re-(N, O) bond distances are ∼2.22 and ∼2.18 Å, respectively, and the Re-P bond distance is the longest in the coordination sphere (∼2.39 Å). The *cis*-angles in the coordination sphere are in the range ∼73–101° and the trans-angles are 169.93(17), 174.01(18), and 178.77(16)°. The five-membered ring in the coordination sphere, defined by Re-N-C-C-O, is almost planar with the largest deviation ∼0.05 Å for C(4).

**Figure 2:**
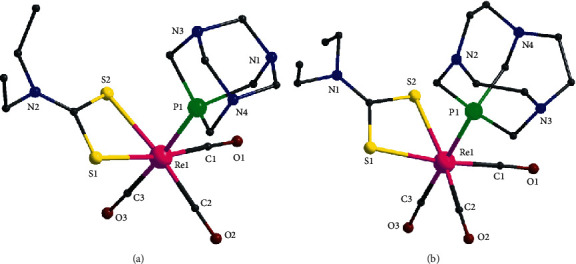
Partially labelled plots of **3a** (a) and **3b** (b).

The molecular structures of **3a** and **3b** are shown in Figure 2; selected bond distances and angles are listed in Table 4. Both complexes consist of the *fac*-[Re^I^(CO)_3_] moiety which is bound to the (S, S) chelate bidentate ligand (diethyldithiocarbamate) and the phosphorus atom of one PTA or CAP ligand (**3a** or **3b**). The coordination geometry around the Re^I^ ion is distorted octahedral in both complexes. There are two short Re-CO bonds in both **3a** and **3b** with bond distances ∼1.91–1.92 Å which are in the *trans*-position with the two sulfur atoms of the diethyldithiocarbamate ligand. The longer Re-CO bond with length ∼2.44–2.46 Å is in the *trans*-position to the phosphorous atom of PTA or CAP. The two Re-S bonds are the longest in the coordination sphere ∼2.52 Å in both complexes. The *cis*-angles in the coordination sphere are in the range ∼69–102^o^ and the trans-angles are in the range 170–179°. The four-membered ring in the coordination sphere, defined by Re-S-C-S atoms, is essentially planar in both complexes with the carbon atom lying ∼0.02 and ∼0.05 Å out of the best mean plane of the four atoms.

Comparing the Re-P bond length between the complexes synthesized, the following trends can be noted: (i) the Re-P bond length between PTA and CAP complexes that carry the same bidentate ligand (**3a** and **3b**) does not differ significantly, i.e., 2.4375 for **3a** and 2.4584 for **3b**; (ii) the Re-P bond length of the PTA complex **3a** is similar to other *fac*-[Re(CO)_3_(bid)PTA] complexes reported in the literature [6, 30]; and (iii) the Re-P bond length of all PTA and CAP complexes synthesized herein is consistently shorter compared to other *fac*-[Re(CO)_3_(bid)P] reported in the literature where P is a phosphine other than PTA and CAP, e.g., PPh_3_, P(Cy)_3_, P(Cy)_2_Ph [6, 30, 43] which is possibly attributed to the better *σ*-donating properties of PTA and CAP.

**Figure 3:**
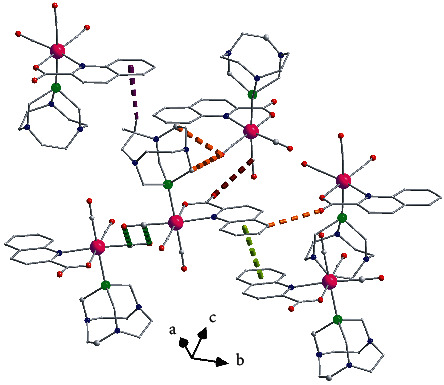
Intermolecular interactions observed in the structure of compound **1b**. Dashed thick dark green (antiparallel C_3_-O_3_⋯C_3_-O_3_) and dark red lines indicate carbonyl-carbonyl interactions (C-O_5_⋯C_1_-O_1_). Dark violet dashed lines indicate C_19_-H_19_⋯*π* interactions. Light green dashed lines indicate *π*⋯*π* between quinaldic ligands and orange dashed lines indicate C_18_-H_18_A⋯O_2_, C_14_-H_14_B⋯O_2_ and C_9_-H_9_⋯O_5_ hydrogen bonds.

**Figure 4:**
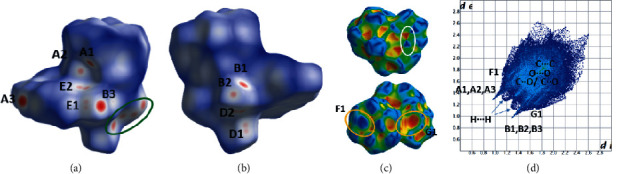
(a), (b) Different views of the dnorm decorated HS, (c) two views of the shape decorated HS, and (d) fingerprint plot for the **1b** complex. A1, A2, A3 and B1, B2, B3 are the donor and acceptor contact points of C_18_-H_18_(A)⋯O_2_, C_14_-H_14_(B)⋯O_2_, C_9_-H_9_⋯O_5_ hydrogen bond interactions. The green ellipse in (a) indicates the area of antiparallel CO⋯CO, C_3_≡O_3_ carbonyl interactions. E1, E2 and D1, D2 are the donor and acceptor contact points involved in the M-C⋯CO, O_5_⋯C1‴ type of interactions. The white and orange ellipses in (c) indicate *π*⋯*π* and C-H⋯*π* (F1 and G1 are donor and acceptor contact points, respectively, Table 5) type of interactions.

The intermolecular interactions in the structures of **1b**, **2b**, **3a,** and **3b** present interesting characteristics and their geometrical characteristics are listed in Table 5 for all structures. These interactions are also studied by using HS analysis tools.  Figure 3(a) presents the intermolecular interactions observed in the structure of compound **1b** among the complexes. In addition to commonly observed hydrogen bond, C-H⋯*π* (Figure 3, Table 5), and *π*⋯*π* interactions [the distance between neighboring centrosymmetrically related quinaldic ligands is 3.467(8) Å, symmetry code (v): −*x*, 1 − *y*, 1 − *z*, Figure 3], the lp(O)-*π* type interactions between carbonyls coordinated to transition metals [39, 57] are also observed. The C_3_≡O_3_ carbonyls are involved in antiparallel CO⋯CO interactions [57], C_3_⋯O_3_^vi^ distance [=C_3_^vi^∙∙∙O_3_, symmetry code (vi): −*x*,−*y*, 1 − *z*] equals to 3.104(5)Å, which is less than the sum (=3.22 Å) of van der Waals(vdW) of C (=1.7 Å) and O (=1.52) atoms] and M-C∙∙∙CO type with the distance O_5_⋯C_1_^i^ and the angle O_5_-C_1_^i^-O_1_^i^ (symmetry code(i): *x*, 0.5−y, 0.5 + *z*) equal to 3.157(4) Å and 70.8(2)° which are close to the usual one [39]. Through these intermolecular interactions, a layer of complexes is formed parallel to the (100) crystallographic plane (Figure S1a). These layers are stacked parallel to [100] crystallographic direction interacting through C-H⋯*π* interactions (Table 5, Figure S1b). The fingerprint plot calculated from HS of complex **1b** is presented in Figure 4(d) where the contribution of each type of intermolecular interactions is indicated. The percentage contribution of the different type of interactions H⋯H, O⋯H/H⋯O, C⋯H/H⋯C, C⋯O/O⋯C, O⋯O, C⋯C, N⋯H/H⋯N are 42.4, 30.3, 12.5, 5.2, 3.7, 2.9, and 2.9%, respectively. All types of interactions discussed above are also clearly seen on the *d*_norm_ HS representation (Figures 4(a) and 4(b)). On the Shape decorated HS, the characteristic blue and red triangles of *π*⋯*π* interactions and the complementary red (concave) and blue (convex) areas characteristic of C-H⋯*π* interactions are present (Figure 4(c).

**Figure 5:**
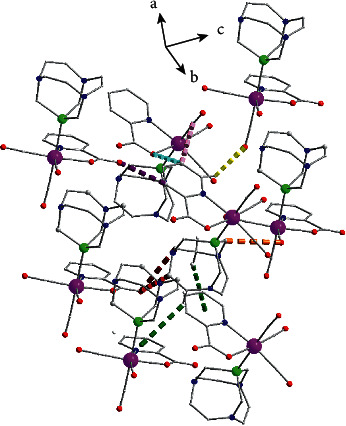
Intermolecular interactions among neighboring clusters in the structure of compound **2b**. The different type of hydrogen bond interactions is indicated with dashed thick orange (C_12_-H_12_⋯O_2_), dark red (C_14_-H_14_A⋯O_5_ and C_15_-H_15_B⋯O_5_ pairs of interactions), and cyan (C_8_-H_8_⋯O_4_) dashed lines. The pink dashed lines indicate carbonyl C_8_-H_8_⋯C_1_ type of interactions. C_6_-H_6_⋯O_5_, C_9_-H_9_⋯O_1_ and C_16_-H_16_∙∙∙Cg_2_ (C-H⋯*π* type of interactions) are indicated with violet, yellow, and dark green dashed lines, respectively.

**Figure 6:**
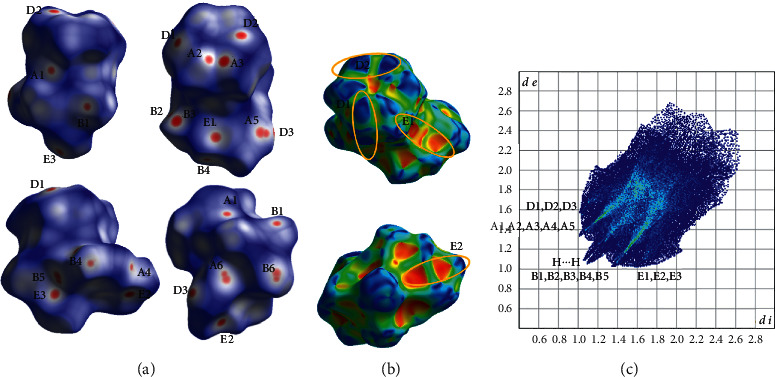
(a) Different views of the *d*_norm_ decorated HS, (b) two views of the shape decorated HS and (c) fingerprint plot for the **2b** complex. A1, A2, A3, A4, A5, A6 and B1, B2, B3, B4, B5, B6 are the donor and acceptor contact points of C_12_—H_12_B⋯O_2_, C_14_—H_14_A⋯O_5_, C_15_—H_15_A⋯O_5_, C_6_—H_6_⋯O_5_, C_8_—H_8_⋯O_4_ and C_9_—H_9_⋯O_1_ hydrogen bond interactions. E1, E2 and D1, D2 are the donor and acceptor contact points involved in the C_13_-H_13_⋯Cg_2_ and C_16_-H_16_···Cg_2_ C-H⋯*π* type of interactions. E3 and D3 are the donor and acceptor contact points of C_8_-H_8_⋯C_1_ type of interactions.

In the structure of compound **2b**, an extensive network of intermolecular interactions is observed such as hydrogen bond and C-H⋯*π* type (Table 5, Figure 5). Layers of complexes are formed parallel to the (100) plane through the hydrogen bond C_12_-H_12_B⋯O_2_, C_14_-H_14_A⋯O_5_, C_15_-H_15_B⋯O_5_, C_8_-H_8_⋯O_4_ and C-H⋯*π* type of interactions, i.e., C_13_-H_13_⋯Cg_2_, C_16_-H_16_⋯Cg_2_ (Figure S2a, Table 5). C-H carbonyl type of interactions is also contributing in the formation of these layers (C_8_-H_8_⋯C1, Figure 5, S2a, Table 5). Neighboring layers are stacked along the a crystallographic axis interacting through hydrogen bond C_6_-H_6_⋯O_5_, C_9_-H_9_⋯O_1_ and C-H C-H∙∙∙*π* type of interactions, i.e., C_16_-H_16_∙∙∙Cg_2_ (Figure S2b, Table 5). The percentage contribution of H⋯H, O⋯H/H⋯O, C⋯H/H⋯C, N⋯H/H⋯N, O⋯O, C⋯O/O⋯C contacts, based on the fingerprint plot analysis (Figure 6(c)) are 32.9, 45.6, 14.0, 2.6, 2.2, and 1.9%, respectively and the contact points of the most characteristic interactions are indicated in *d*_norm_ decorated HS in Figure 6(a). C-H∙∙∙*π* interactions reveal their presence in the characteristic complementary red (concave) and blue (convex) areas on the HS decorated with Shape property (Figure 6(b)).

**Figure 7:**
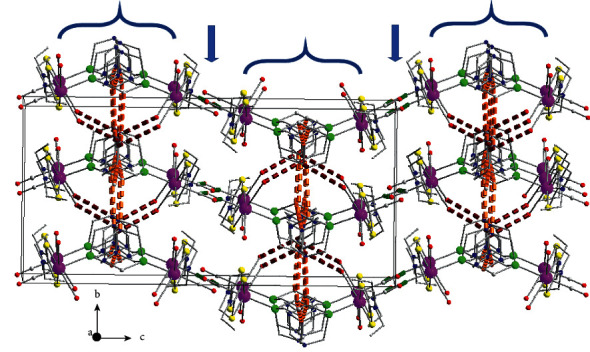
Stacking of layers along the c crystallographic axis in the structure of compound **3a**. Braces indicate the position of layers and arrows the carbonyl-carbonyl interactions. The dashed thick orange lines indicate C_9_-H_9_A⋯N_4_ and C_9_-H_9_B⋯N_3_ and the dark red one indicates C_13_-H_13_A⋯O_1_ hydrogen bonds. Dashed thick dark green lines indicate antiparallel CO⋯CO carbonyl-carbonyl interactions. Details for the arrangement of complexes and the intermolecular interactions within the layer are presented in Figure S3.

**Figure 8:**
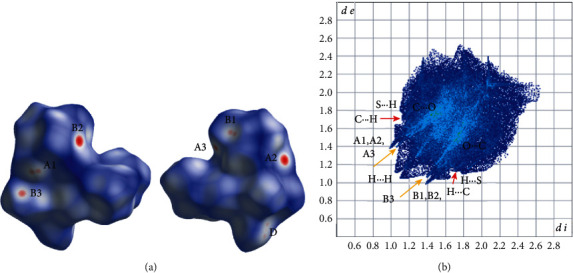
(a), Different views of the *d*_norm_ decorated HS and (b) fingerprint plot for the **3a** complex. A1, A2, A3 and B1, B2, B3 are the donor and acceptor contact points of C_9_—H_9_(A)⋯N_4_, C_9_—H_9_(B)⋯N_3_ and C_13_—H_13_A⋯O_1_(indicated with orange arrows in fingerprint plot) hydrogen bond interactions (Table 5). Label D indicates the area of antiparallel CO⋯CO, C_3_≡O_3_ carbonyl interactions. Red arrows in the fingerprint plot indicate the C⋯H/⋯C contact points.

In the structure of **3a**, layers of complexes are formed parallel to the (001) plane through hydrogen bond interactions (Figure S3, Table 5). These layers are stacked along the *c* crystallographic axis (Figure 7) and complexes belonging to neighboring layers interact through antiparallel CO⋯CO interactions, where the C_3_≡O_3_ carbonyls are involved (C_3_⋯O_3_*∗* distance is equal to 3.180(4)Å). The percentage contribution of H⋯H, O⋯H/H⋯O, S⋯H/H⋯S, N⋯H/H⋯N, C⋯H/H⋯C, C⋯O/O⋯C, C⋯S/S⋯C, O⋯S/S⋯O contacts derived from fingerprint plot analysis (Figure 8(b)) are 38.0, 31.5, 8.6, 7.8, 7.1, 2.3, 1.4, and 1.0%, respectively. The most intense interactions, i.e., C9-H9A∙∙∙N4, C9-H9AB∙∙N3 and C13-H13A∙∙∙O1 appear on the *d*_norm_ decorated HS in Figure 8(a).

**Figure 9:**
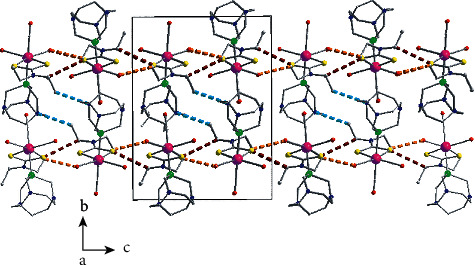
Stacking of layers along the b crystallographic axis in the structure of compound **3b**, The dashed thick orange, dark red, and cyan lines indicate C_8_-H_8_A⋯O_2_, C_5_-H_5_⋯S_2_ and C_6_-H_6_B⋯N_2_ hydrogen bonds, respectively. Details for the arrangement of complexes and the intermolecular interactions within the layer are presented in Figure S4.

**Figure 10:**
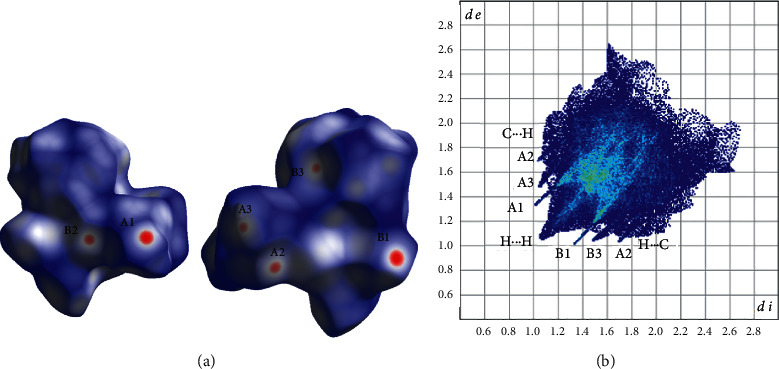
(a) Different views of the *d*_norm_ decorated HS and (b) fingerprint plot for the **3b** complex. A1, A2, A3 and B1, B2, B3 are the donor and acceptor contact points of C_8_-H_8_A⋯O_2_, C_5_-H_5_A∙∙S_2_ and C_6_-H_6_B⋯N_2_ hydrogen bond interactions (Table 5).

In the case of **3b**, complexes interacting through the C_8_-H_8_A⋯O_2_ and C_5_-H_5_A⋯S_2_ hydrogen bonds (Table 5) form layers parallel to the plane (010) (Figure S4) and through the C_6_-H_6_B⋯N_2_ interactions of complexes belonging to neighboring layers stacked along the *b* axis, the 3D architecture of the structure is built (Figure 9). The percentage contribution of H⋯H, O⋯H/H⋯O, C⋯H/H⋯C, S⋯H/H⋯S, N⋯H/H⋯N contacts derived from fingerprint plot analysis (Figure 10(b)) are 49.2, 29.7, 7.7, 7.0, and 2.5, respectively. Each one of C⋯O/O⋯C, C⋯S/S⋯C, O⋯S/S⋯O type of contacts contributes less than 1%. The most intense interactions, i.e., C_8_-H_8_A⋯O_2_, C_5_-H_5_A⋯S_2_ and C_6_-H_6_B⋯N_2_ appear on the *d*_norm_ decorated HS in Figure 10(a), and as spikes in the fingerprint plot (Figure 10(b)).

## 4. Conclusions

In this work, the coordination chemistry of rhenium with the hydrophilic monodentate phosphines PTA and CAP was explored by synthesizing and characterizing a series of novel [2 + 1] mixed-ligand *fac*-[Re(CO)_3_(bid) (X)] complexes. Both PTA and CAP serve the role of the polar and hydrophilic modifier aiming to develop a new platform of hydrophilic *fac*-[Re(CO_3_] complexes with favorable pharmacokinetics. The detailed crystal structure studies using the Hirshfeld surface analysis tools have revealed that the C-H⋯O type of intermolecular interactions has the largest contribution in the packing of complexes. In the case of **1b, ***π*⋯*π* are one type of the characteristic intermolecular interactions that contribute to the packing of complexes and for the rest are the C-H⋯*π*. In all structures, the PTA and CAP ligands are coordinated through the P atom with Re and the observation of N···H type of interactions in all studied structures reveals the potential of these three nitrogen atoms to develop hydrogen bonds with their environment and thus to impact the pharmacokinetic behavior of these compounds. In the case of **1b** and **3b**, carbonyl-carbonyl intermolecular interactions are observed among the complexes, which is a type of interaction which recently is discussed as a potential path which could impact the biological and physical properties of these compounds.

This system could potentially be applied in (radio) pharmaceutical design to develop complexes with suitable properties for diagnosis (^99m^Tc), radiotherapy (^186/188^Re), and chemotherapy (^185/187^Re) by tethering a biologically active molecule either to the bidentate or the phosphine ligand. The transfer of the coordination chemistry at the ^99m^Tc level, the evaluation of the hydrophilicity of the corresponding complexes, and the investigation of its *in vivo* performance are currently in progress.

## Figures and Tables

**Table 1:** Crystallographic data for complexes **1b**, **2b**, **3a,** and **3b**.

	**1b**	**2b**	**3a**	**3b**
Formula	C_22_H_24_N_4_O_5_PRe	C_18_H_22_N_4_O_5_PRe	C_14_H_22_N_4_O_3_PReS_2_	C_17_H_28_N_4_O_3_PReS_2_
*Fw*	641.62	591.56	575.64	617.72
Space group	*P*2_1_/*c*	*P*2_1_	*P*bca	*P*2_1_/*c*
*a* (Å)	10.8032 (5)	10.4241 (6)	11.9639 (3)	11.0443 (2)
*b* (Å)	14.8257 (7)	7.4583 (4)	12.5031 (3)	16.2008 (3)
*c* (Å)	14.4608 (7)	13.0408 (8)	26.2270 (7)	12.6720 (2)
*α* (°)	90.0	90.0	90.0	90.0
*β* (°)	98.365 (2)	98.365 (2)	90.0	103.095 (1)
*γ* (°)	90.0	90.0	90.0	90.0
*V* (Å^3^)	2291.47 (19)	1003.08 (10)	3923.19 (17)	2208.40 (7)
*Z*	4	2	8	4
*T* (°C)	20	−113	20	−113
Radiation	Mo K*α*	Mo K*α*	Mo K*α*	Mo K*α*
*ρ*_calcd_ (g cm^−3^)	1.860	1.959	1.949	1.858
*μ* (mm^−1^)	5.415	6.175	6.510	5.789
2*θ*_max_	54.0°	54.0°	54.0°	54.0°
Reflections collected/unique/used	53018/5011/5011	18362/4349/4349	32531/4253/4253	41273/4812/4812
*R*_int_	0.0371	0.0252	0.0518	0.0299
Parameters refined	346	278	292	365
Reflections with *I* > 2*σ* (I)	4501	4249	3688	4421
*R*_1_^a^/*wR*_2_^a^[*I* > 2*σ* (I)]	0.0214/0.0461	0.0173/0.0401	0.0231/0.0554	0.0173/0.0358
*R*1^a^/w*R*2^a^ (for all data)	0.0252/0.0471	0.0178/0.0403	0.0284/0.0578	0.0201/0.0365
(Δ/*σ*)_max_	0.002	0.003	0.001	0.002
(Δ*ρ*)_max_/ (Δ*ρ*)_min_ (e/Å^3^)	0.828/−0.800	0.721/−0.443	0.727/−0.787	0.749/−0.636

^a^ *w*=1/[*σ*^2^(*F*_*o*_^2^)+(*αP*))^2^+*bP*] and *P*=[max(*F*_*o*_^2^, 0)+2*F*_*c*_^2^]/3, *a* = 0.0192, *b* = 2.3883 (**1b**);*a* = 0.0134, *b* = 0.5262 (**2b**); *a* = 0.0281, *b* = 0.7043 (**3a**); *a* = 0.0143, *b* = 1.7692 (**3b**); *R*_1_ = ∑(|*F*_*o*_| − |*F*_*c*_|)/∑(|*F*_*o*_|) and *wR*_2_ ={∑[*w*(*F*_*o*_^2^ − *F*_*c*_^2^)^2^]/∑[*w*(*F*_*o*_^2^)^2^]}0^1/2^.

**Table 2:** ^31^P and ^13^C (C ≡ O) NMR chemical shifts for PTA, CAP and all Re complexes^a^.

	PTA	CAP	**1a**	**1b**	**2a**	**2b**	**3a**	**3b**
^**31**^ **P**	−104.01	47.08	−71.65	38.66	−73.26	37.21	−84.56	29.17
^**13**^ **C (C≡O)**	—	—	194.59 (cis)	195.76 (cis)	194.78 (cis)	196.13 (cis)	191.83 (cis)	193.16 (cis)
			(*J* = 8.1 Hz)	(*J* = 5.7 Hz)	(*J* = 7.6 Hz)	(*J* = 5.1 Hz)	(*J* = 8.2 Hz)	(*J* = 6.7 Hz)
			194.44 (cis)	190.94 (trans)	193.94 (cis)	195.23 (cis)	190.26 (trans)	191.23 (trans)
			(*J* = 8.1 Hz)	(*J* = 58.3 Hz)	(*J* = 7.6 Hz)	(*J* = 5.1 Hz)	(*J* = 69.9 Hz)	(*J* = 57.4 Hz)
			189.62 (trans)		190.03 (trans)	191.38 (trans)		
			(*J* = 70.1 Hz)		(*J* = 70.8 Hz)	(*J* = 59.1 Hz)		

^a^All spectra are recorded in DMSO-*d*_6_, *J* refers to ^2^*J*_C-P_, and chemical shifts are in ppm.

**Table 3:** Selected bond distances (Å) and angles (°) for **1b** and **2b**.

	**1b**	**2b**
*Distances*		
Re (1)-C (2)	1.889 (3)	1.939 (5)
Re (1)-C (3)	1.907 (3)	1.938 (5)
Re (1)-C (1)	1.965 (3)	1.881 (6)
Re (1)-O (4)	2.140 (2)	2.184 (3)
Re (1)-N (1)	2.244 (2)	2.223 (4)
Re (1)-P (1)	2.4543 (8)	2.3910 (12)

*Angles*		
C (2)-Re (1)-C (3)	83.99 (14)	88.5 (2)
C (2)-Re (1)-C (1)	91.24 (13)	89.6 (2)
C (3)-Re (1)-C (1)	90.66 (14)	90.7 (2)
C (2)-Re (1)-O (4)	179.34 (12)	96.70 (17)
C (3)-Re (1)-O (4)	95.93 (12)	174.01 (18)
C (1)-Re (1)-O (4)	89.42 (11)	92.27 (19)
C (2)-Re (1)-N (1)	105.16 (11)	169.93 (17)
C (3)-Re (1)-N (1)	170.84 (12)	101.50 (19)
C (1)-Re (1)-N (1)	89.65 (11)	91.5 (2)
O (4)-Re (1)-N (1)	74.92 (8)	73.25 (13)
C (2)-Re (1)-P (1)	92.62 (10)	89.88 (16)
C (3)-Re (1)-P (1)	87.63 (11)	88.13 (16)
C (1)-Re (1)-P (1)	175.59 (9)	178.77 (16)
O (4)-Re (1)-P (1)	86.73 (7)	88.90 (10)
N (1)-Re (1)-P (1)	91.40 (6)	89.24 (10)

**Table 4:** Selected bond distances (Å) and angles (°) for **3a** and **3b**.

	**3a**	**3b**
*Distances*		
Re (1)-C (1)	1.911 (3)	1.923 (3)
Re (1)-C (2)	1.920 (3)	1.905 (3)
Re (1)-C (3)	1.952 (3)	1.955 (3)
Re (1)-P (1)	2.4375 (7)	2.4584 (6)
Re (1)-S (2)	2.5069 (8)	2.5245 (6)
Re (1)-S (1)	2.5232 (8)	2.5236 (6)

*Angles*		
C (1)-Re (1)-C (2)	87.90 (14)	89.43 (11)
C (1)-Re (1)-C (3)	90.63 (14)	91.10 (11)
C (2)-Re (1)-C (3)	92.42 (13)	89.04 (11)
C (1)-Re (1)-P (1)	92.11 (10)	88.73 (8)
C (2)-Re (1)-P (1)	91.48 (9)	91.91 (8)
C (3)-Re (1)-P (1)	175.32 (10)	179.02 (8)
C (1)-Re (1)-S (2)	101.80 (10)	101.26 (7)
C (2)-Re (1)-S (2)	170.30 (10)	168.15 (8)
C (3)-Re (1)-S (2)	87.48 (10)	95.77 (8)
P (1)-Re (1)-S (2)	88.25 (2)	83.32 (2)
C (1)-Re (1)-S (1)	171.10 (10)	171.03 (7)
C (2)-Re (1)-S (1)	100.42 (10)	99.50 (8)
C (3)-Re (1)-S (1)	92.16 (10)	89.90 (8)
P (1)-Re (1)-S (1)	84.58 (3)	90.12 (2)
S (2)-Re (1)-S (1)	69.89 (3)	69.764 (19)

**Table 5:** Hydrogen-bond geometry (Å, °) for **1b**, **2b**, **3a,** and **3b**.

D—H⋯A	D—H (Å)	H⋯A (Å)	D⋯A (Å)	D—H⋯A (°)	Symmetry operation
**Complex-1b**					
C_18_—H_18_A⋯O_2_^i^	0.97	2.49	3.421 (5)	161	(i): *x*, −*y* + 0.5, *z* + 0.5
C_14_—H_14_B⋯O_2_^ii^	0.96 (5)	2.59 (5)	3.470 (5)	151 (4)	(ii): *x*, −*y* + 1/2, *z* + 1/2
C_9_—H_9_⋯O_5_^iii^	0.85 (3)	2.48 (3)	3.308 (5)	162 (3)	(iii): −*x*, 0.5 + *y*, 1.5 − *z*
C_19_—H_19_⋯Cg_1_^iv^ (Cg_1_: centroid of C_8_ C_13_ phenyl ring)	0.97 (1)	3.009 (1)	3.887 (5)	151.3 (3)	(iv): 1 − *x*, −0.5 + *y*, 1.5 − *z*

**Complex-2b**					
C_12_—H_12_B⋯O_2_^i^	0.99	2.55	3.434 (9)	149	(i):−*x* + 1, *y* − 1/2
C_14_—H_14_A⋯O_5_^ii^	0.99	2.56	3.521 (9)	164	(ii): −*x* + 1, *y* − 1/2,−*z* + 1
C_15_—H_15_B⋯O_5_^iii^	0.99	2.46	3.411 (9)	162	(iii): −*x* + 1, *y* − 1/2,−*z* + 1
C_6_—H_6_⋯O_5_^iv^	0.97 (5)	2.56 (5)	3.210 (7)	125 (4)	(iv): 2 − *x*, −0.5 + *y*, 1 − *z*
C_8_—H_8_⋯O_4_^v^	0.77 (6)	2.62 (6)	3.079 (7)	120 (5)	(v): *x*, −1 + *y*, *z*
C_9_—H_9_⋯O_1_^vi^	1.05 (8)	2.50 (7)	3.104 (7)	116 (5)	(vi): 2 − *x*,−0.5 + *y*, 2 −*z*
C_8_—H_8_⋯C_1_^vii^	0.77 (6)	2.92 (6)	3.682 (9)	170 (5)	(vii): *x*,−1 + *y*, *z*
C_13_—H_13_⋯Cg_2_^viii^	0.99 (1)	3.4366 (2)	4.154 (9)	130.0 (4)	(viii): 1 − *x*, 0.5 + *y*, 1 − *z*
C_16_—H_16_⋯Cg_2_^ix^ (Cg_2_: Centroid of N_1_, C_5_…C_9_ pyridine ring)	0.99 (1)	3.2873 (2)	4.211 (1)	156.0 (6)	(i*x*): −1 + *x*, *y*, *z*

**Complex-3a**					
C_9_—H_9_A⋯N_4_^i^	0.97 (4)	2.68 (4)	3.627 (4)	166 (2)	(i) −*x* + 1/2, *y* − 1/2, *z*
C_9_—H_9_B⋯N_3_^ii^	0.94 (4)	2.53 (4)	3.413 (4)	158 (3)	(ii) *x* − 1/2, *y*,−*z* + 1/2
C_13_—H_13_A⋯O_1_^iii^	1.00 (3)	2.56 (3)	3.534 (5)	165 (3)	(iii) −*x* + 1/2, *y* + 1/2

**Complex-3b**					
C_5_—H_5_A⋯S_2_^i^	1.02 (3)	2.80 (3)	3.701 (3)	147 (2)	(i): *x*, −*y* + 1/2, *z* − 1/2
C_8_—H_8_A⋯O_2_^iii^	0.97 (4)	2.48 (4)	3.434 (4)	168 (3)	(ii) −*x* + 1, −*y* + 1, −*z* + 1
C_6_—H_6_B⋯N_2_^ii^	0.95 (4)	2.67 (4)	3.580 (4)	160 (3)	(iii) *x* + 1, −*y* + 1/2, *z* + 1/2

## Data Availability

Crystallographic data for the structures reported in this manuscript have been deposited with the Cambridge Crystallographic Data Centre under the CCDC numbers: 2152941 (compound **1b**), 2152938 (compound **2b**), 2152939 (compound **3a**), and 2152940 (compound **3b**). Copies of these data can be obtained free of charge from https://www.ccdc.cam.ac.uk/data_request/cif.

## Supplementary Materials

Supplementary Materials Figure S1: Intermolecular interactions in the structure of compound **1b** forming (a) layers of complexes parallel to the crystallographic plane (100) and (b) Stacks of layers along the a crystallographic axis. Figure S2: (a) Intermolecular interactions among neighboring clusters forming layers of complexes arranged parallel to the crystallographic plane (100), and (b) stacking of layers along the a crystallographic axis in the structure of compound **2b** through hydrogen bond, C-H∙∙∙*π* type, and carbonyl interactions. Figure S3: Layers of complexes arranged parallel to the crystallographic plane (001) in the structure of compound **3a** through hydrogen bond interactions. Figure S4: Layers of complexes arranged parallel to the crystallographic plane (010) in the structure of compound **3b**.

## References

[1] Lepareur N., Lacœuille F., Bouvry C. (2019). Rhenium-188 labeled radiopharmaceuticals: current clinical applications in oncology and promising perspectives. *Frontiers of Medicine*.

[2] Abram U., Alberto R. (2006). Technetium and rhenium: coordination chemistry and nuclear medical applications. *Journal of the Brazilian Chemical Society*.

[3] Konkankit C. C., Marker S. C., Knopf K. M., Wilson J. J. (2018). Anticancer activity of complexes of the third row transition metals, rhenium, osmium, and iridium. *Dalton Transactions*.

[4] Savjani K. T., Gajjar A. K., Savjani J. K. (2012). Drug solubility: importance and enhancement techniques. *ISRN Pharmaceutics*.

[5] Giammei C., Balber T., Bencurova K. (2020). Sorbitol as a polar pharmacological modifier to enhance the hydrophilicity of Tc-99m-tricarbonyl-based radiopharmaceuticals. *Molecules*.

[6] Gantsho V. L., Dotou M., Jakubaszek M. (2020). Synthesis, characterization, kinetic investigation and biological evaluation of Re(I) di- and tricarbonyl complexes with tertiary phosphine ligands. *Dalton Transactions*.

[7] Römhild K., Fischer C. A., Mindt T. L. (2017). Glycated 99m Tc-tricarbonyl-labeled peptide conjugates for tumor targeting by “click-to-chelate”. *ChemMedChem*.

[8] Britvin S. N., Lotnyk A. (2015). Water-soluble phosphine capable of dissolving elemental gold: the missing link between 1,3,5-triaza-7-phosphaadamantane (PTA) and verkade’s ephemeral ligand. *Journal Of the American Chemical Society*.

[9] Andrew L. G., Phillips D., Romerosa A., Vizza F., Peruzzini M. (2004). Coordination chemistry of 1,3,5-triaza- 7-phosphaadamantane (PTA): transition metal complexes and related catalytic, medicinal and photoluminescent applications. *Coordination Chemistry Reviews*.

[10] Guerriero A., Gonsalvi L. (2021). From traditional PTA to novel CAP: a comparison between two adamantane cage-type aminophosphines. *Inorganica Chimica Acta*.

[11] Miroslavov A. E., Britvin S. N., Braband H. (2019). Water-soluble carbonyl complexes of 99Tc(I) and Re(I) with adamantane-cage aminophosphines PTA and CAP. *Journal of Organometallic Chemistry*.

[12] Scattolin T., Voloshkin V. A., Martynova E. (2021). Synthesis and catalytic activity of palladium complexes bearing N-heterocyclic carbenes (NHCs) and 1,4,7-triaza-9-phosphatricyclo[5.3.2.1]tridecane (CAP) ligands. *Dalton Transactions*.

[13] Guerriero A., Peruzzini M., Gonsalvi L. (2018). Coordination chemistry of 1,3,5-triaza-7-phosphatricyclo[3.3.1.1]decane (PTA) and derivatives. Part III. Variations on a theme: novel architectures, materials and applications. *Coordination Chemistry Reviews*.

[14] Bravo J., Bolaño S., Gonsalvi L., Peruzzini M. (2010). Coordination chemistry of 1,3,5-triaza-7-phosphaadamantane (PTA) and derivatives. Part II. The quest for tailored ligands, complexes and related applications. *Coordination Chemistry Reviews*.

[15] Murray B. S., Babak M. V., Hartinger C. G., Dyson P. J. (2016). The development of RAPTA compounds for the treatment of tumors. *Coordination Chemistry Reviews*.

[16] Scalambra F., Lorenzo‐Luis P., de los Ríos I., Romerosa A. (2019). New findings in metal complexes with antiproliferative activity containing 1,3,5-triaza-7-phosphaadamantane (PTA) and derivative ligands. *European Journal of Inorganic Chemistry*.

[17] Sgarbossa P., Sliwinska-Hill U., Guedes da Ilva M. F. C. (2019). Pentafluorophenyl platinum(II) complexes of, 3907 of PTA and its N-allyl and N-benzyl derivatives: synthesis, characterization and biological activity. *Materials*.

[18] Alshamrani A. F., Prior T. J., Burke B. P. (2020). Water-Soluble rhenium phosphine complexes incorporating the Ph_2_C(X) motif (X = O-, NH-): structural and cytotoxicity studies. *Inorganic Chemistry*.

[19] Bolaño S., Gonsalvi L., Barbaro P. (2006). Synthesis, characterization, protonation studies and X-ray crystal structure of ReH_5_(PPh_3_)_2_(PTA) (PTA=1,3,5-triaza-7-phosphaadamantane). *Journal Of Organometallic Chemistry*.

[20] Bordoloi J. K., Berry D., Khan I. U. (2015). Technetium-99m and rhenium-188 complexes with one and two pendant bisphosphonate groups for imaging arterial calcification. *Dalton Transactions*.

[21] Maccaroni E., Dong H., Blacque O., Schmalle H. W., Frech C. M., Berke H. (2010). Water soluble phosphine rhenium complexes. *Journal Of Organometallic Chemistry*.

[22] Marchi A., Marchesi E., Marvelli L., Bergamini P., Bertolasi V., Ferretti V. (2008). New water-soluble rhenium complexes with 1,3,5-triaza-7-phosphaadamantane (PTA)—X-ray crystal structures of [ReNCl_2_ (PTA) 3], [ReO_2_ Cl(PTA) 3], [ReCl 3 -(PTA) 2 (PPh 3)], and [Re_2_ N_2_ Cl_3_ (Et_2_ dtc)-(PTA) 4]. *European Journal of Inorganic Chemistry*.

[23] Martins L. M. D. R. S., Alegria E. C. B. A., Smoleński P., Kuznetsov M. L., Pombeiro A. J. L. (2013). Oxorhenium complexes bearing the water-soluble tris(pyrazol-1-yl)methanesulfonate, 1,3,5-triaza-7-phosphaadamantane, or related ligands, as catalysts for Baeyer-Villiger oxidation of ketones. *Inorganic Chemistry*.

[24] Marvelli L., Bergamini P., Marchi A., Bersani G., Ferretti V., Bertolasi V. (2018). Direct formation of new water soluble Re and Tc complexes containing PTA (1,3,5-triaza-7-phosphaadamantane) from their permetallated salts. Reactivity and X-ray crystal structures. *Inorganica Chimica Acta*.

[25] Shan X., Ellern A., Espenson J. H. (2002). Methyl transfer from rhenium to coordinated thiolate groups. *Angewandte Chemie*.

[26] Smoleński P., Pombeiro A. J. L. (2008). Water-soluble and stable dinitrogen phosphine complexes trans-[ReCl(N_2_)(PTA-H)n(PTA)4−n]n + (n = 0-4), the first with 1,3,5-triaza-7-phosphaadamantane. *Dalton Transactions*.

[27] Schibli R., Katti K. V., Volkert W. A., Barnes C. L. (1998). Novel coordination behavior of fac-[ReBr_3_(CO)_3_]_2_- with 1,3,5-triaza-7-phosphaadamantane (PTA). Systematic investigation on stepwise replacement of the halides by PTA ligand. Phase transfer studies and X-ray crystal structure of [NEt4][ReBr_2_((PTA)(CO)_3_], [ReBr(PTA)_2_(CO)_3_], and [Re(PTA)_3_(CO)_3_]PF_6_. *Inorganic Chemistry*.

[28] Chakraborty I., Carrington S. J., Roseman G., Mascharak P. K. (2017). Synthesis, structures, and CO release capacity of a family of water-soluble PhotoCORMs: assessment of the biocompatibility and their phototoxicity toward human breast cancer cells. *Inorganic Chemistry*.

[29] Herrick R. S., Ziegler C. J., Sripothongnak S. (2009). Preparation and characterization of rhenium (I) tricarbonyl dithiocarbamate compounds; Re(CO)_3_(S_2_CNMe_2_)(L). *Journal of Organometallic Chemistry*.

[30] Manicum A., Alexander O., Schutte-Smith M., Visser H. G. (2020). Synthesis, characterization and substitution reactions of fac-[Re(O,O′-bid)(CO)_3_(P)] complexes, using the “2 + 1” mixed ligand model. *Journal Of Molecular Structure*.

[31] Manicum A., Schutte-Smith M., Alexander O. T., Twigge L., Roodt A., Visser H. G. (2019). First kinetic data of the CO substitution in fac-[Re(L,L′-Bid)(CO)_3_(X)] complexes (L,L′-Bid = acacetylacetonate or tropolonate) by tertiary phosphines PTA and PPh3: synthesis and crystal structures of water-soluble rhenium(I) tri- and dicarbonyl complexes with 1,3,5-triaza-7-phosphaadamantane (PTA). *Inorganic Chemistry Communications*.

[32] Marker S. C., MacMillan S. N., Zipfel W. R., Li Z., Ford P. C., Wilson J. J. (2018). Photoactivated in vitro anticancer activity of rhenium(I) tricarbonyl complexes bearing water-soluble phosphines. *Inorganic Chemistry*.

[33] Kia R., Kalaghchi A. (2020). Structural, non-covalent interaction, and natural bond orbital studies on bromido-tricarbonyl rhenium(I) complexes bearing alkyl-substituted 1,4-diazabutadiene (DAB) ligands. *Crystals*.

[34] Kia R., Mahmoudi S., Raithby P. R. (2019). New rhenium-tricarbonyl complexes bearing halogen-substituted bidentate ligands: structural, computational and Hirshfeld surfaces studies. *CrystEngComm*.

[35] Mark-Lee W. F., Chong Y. Y., Kassim M. B. (2018). Supramolecular structures of rhenium(I) complexes mediated by ligand planarity via the interplay of substituents. *Acta Crystallographica Section C Structural Chemistry*.

[36] Ismail M. B., Booysen I. N., Akerman M. P. (2018). Coordination susceptibilities of cinnamaldehyde and cuminaldehyde derived Schiff bases towards the fac-[Re(CO)_3_]+ core: formation, computational and DNA interaction studies. *Inorganica Chimica Acta*.

[37] Christopher Krauss V. S., Smith V., Krauss C. (2013). The effect of novel rhenium compounds on lymphosarcoma, PC-3 prostate and myeloid leukemia cancer cell lines and an investigation on the DNA binding properties of one of these compounds through electronic spectroscopy. *Journal of Bioprocessing & Biotechniques*.

[38] Allen F. H., Baalham C. A., Lommerse J. P. M., Raithby P. R. (1998). Carbonyl-carbonyl interactions can be competitive with hydrogen bonds. *Acta Crystallographica Section B Structural Science*.

[39] Echeverría J. (2018). Intermolecular carbonyl···carbonyl interactions in transition-metal complexes. *Inorganic Chemistry*.

[40] Echeverría J. (2018). The *n π∗* interaction in metal complexes. *Chemical Communications*.

[41] Guerriero A., Oberhauser W., Riedel T., Peruzzini M., Dyson P. J., Gonsalvi L. (2017). New class of half-sandwich ruthenium(II) arene complexes bearing the water-soluble CAP ligand as an in vitro anticancer agent. *Inorganic Chemistry*.

[42] Alberto R., Egli A., Abram U., Hegetschweiler K., Gramlich V., Schubiger P. A. (1994). Synthesis and reactivity of [NEt_4_]_2_[ReBr_3_(CO)_3_]. Formation and structural characterization of the clusters [NEt_4_][Re_3_(*μ*3-OH)(*μ*-OH)_3_(CO)_9_] and [NEt4][Re_2_(*μ*-OH)_3_(CO)_6_] by alkaline titration. *Journal of the Chemical Society Dalton Transactions*.

[43] Triantis C., Shegani A., Kiritsis C. (2018). Dicarbonyl cis-[M(CO)_2_(N, O)(C)(P)] (M = Re, 99mTc) complexes with a new [2 + 1 + 1] donor atom combination. *Inorganic Chemistry*.

[44] Schibli R., La Bella R., Alberto R. (2000). Influence of the denticity of ligand systems on the in vitro and in vivo behavior of 99mTc(I)−tricarbonyl complexes: a hint for the future functionalization of biomolecules. *Bioconjugate Chemistry*.

[45] Rigaku T. W. (2005). *Crystal Clear*.

[46] Sheldrick G. M. (2008). A short history of SHELX. *Acta Crystallographica Section A Foundations of Crystallography*.

[47] Sheldrick G. M. (2015). Crystal structure refinement with SHELXL. *Acta Crystallographica Section C Structural Chemistry*.

[48] Diamond V. (1999). *Crystal and Molecular Structure Visualization, Crystal Impact*.

[49] Spackman P. R., Turner M. J., McKinnon J. J., Wolff S. K., Grimwood D. J., Jayatilaka D. (2021). CrystalExplorer: a program for Hirshfeld surface analysis, visualization and quantitative analysis of molecular crystals. *Journal of Applied Crystallography*.

[50] McKinnon J. J., Spackman M. A., Mitchell A. S. (2004). Novel tools for visualizing and exploring intermolecular interactions in molecular crystals. *Acta Crystallographica Section B Structural Science*.

[51] Papagiannopoulou D., Triantis C., Vassileiadis V. (2014). Synthesis, structural characterization and radiochemistry of di- and tricarbonyl Re(I) and 99mTc(I) complexes with 8-hydroxyquinoline or 8-mercaptoquinoline and triphenylphosphine. *Polyhedron*.

[52] Kyprianidou P., Tsoukalas C., Chiotellis A. (2011). First example of well-characterized Re and 99mTc tricarbonyl complexes of ciprofloxacin and norfloxacin in the development of infection-specific imaging agents. *Inorganica Chimica Acta*.

[53] Karagiorgou O., Patsis G., Pelecanou M. (2005). (S)-(2-(2′-Pyridyl)ethyl)cysteamine and (S)-(2-(2′-Pyridyl)ethyl)-d,l-homocysteine as ligands for the “fac-[M(CO)_3_] +” (M = Re, 99mTc) core. *Inorganic Chemistry*.

[54] Lazopoulos A., Triantis C., Shegani A. (2021). Effective labeling of amine pharmacophores through the employment of 2,3-pyrazinedicarboxylic anhydride and the generation of fac-[M(CO)_3_(PyA)P] and cis-trans-[M(CO)_2_(PyA)P_2_] complexes (PyA = pyrazine-2-carboxylate, P = phosphine, M = Re, 99mTc). *Inorganic Chemistry*.

[55] Ozer Z., Ozkar S. (1999). C-13-and P-31-NMR study of tetracarbonylbis(diphenylphosphino)alkanemetal(0) complexes of the group 6 elements. *Turkish Journal of Chemistry*.

[56] Britvin S. N., Rumyantsev A. M., Zobnina A. E., Padkina M. V. (2016). Between adamantane and atrane: intrabridgehead interactions in the cage-like phosphane related to a novel tris(homoadamantane) ring system. *Chemistry—A European Journal*.

[57] Kia R., Taghavi T., Raithby P. R. (2020). Supramolecular assembly through intermolecular n *π∗* interactions through a coordinated perrhenate formed via superoxidation of Re(i) to Re(vii) in the formation of substituted Re(CO)3 complexes bearing Diimine ligands. *CrystEngComm*.

