# Supplementary material for: Synthesis and Characterization of Novel [2 + 1] Tricarbonyl Rhenium Complexes with the Hydrophilic Phosphine Ligands PTA and CAP
Source: Bioinorg Chem Appl. 2022 Jun 13;2022:3117661. doi: 10.1155/2022/3117661 (PMC9208990; doi:10.1155/2022/3117661)
Supplement: Supplementary Materials — Figure S1: Intermolecular interactions in the structure of compound 1b forming (a) layers of complexes parallel to the crystallographic plane (100) and (b) Stacks of layers along the a crystallographic axis. Figure S2: (a) Intermolecular interactions among neighboring clusters forming layers of complexes arranged parallel to the crystallographic plane (100), and (b) stacking of layers along the a crystallographic axis in the structure of compound 2b through hydrogen bond, C-H∙∙∙π type, and carbonyl interactions. Figure S3: Layers of complexes arranged parallel to the crystallographic plane (001) in the structure of compound 3a through hydrogen bond interactions. Figure S4: Layers of complexes arranged parallel to the crystallographic plane (010) in the structure of compound 3b. [file 3117661.f1.pdf]

Supplementary material for:

Synthesis and characterization of novel [2 + 1] tricarbonyl rhenium complexes with the hydrophilic phosphine ligands PTA and CAP

Ioanna Roupa,[1] Charalambos Flabouraris,[1] Antonio Shegani,[1] Myrto Ischyropoulou,[1] Konstantina Makrypidi,[1] Katerina Raptopoulou,[2] Ioannis Pirmettis,[1] Minas S. Papadopoulos,[1] Vassilis Psycharis[2] and Aristeidis Chiotellis[1,*]

Institutes of [1]Nuclear & Radiological Sciences & Technology, Energy & Safety, [2]Nanoscience and Nanotechnology, National Center for Scientific Research "Demokritos", 15310 Athens, Greece.

Correspondence should be addressed to Aristeidis Chiotellis; achiotel@rrp.demokritos.gr

TABLE OF CONTENTS

Figure S1: Intermolecular interactions in the structure of compound 1b forming a) layers of complexes parallel to the crystallographic plane (100) and (b) Stacks of layers along the a crystallographic axis — S2

Figure S2: (a) Intermolecular interactions among neighbouring clusters forming layers of complexes arranged parallel to the crystallographic plane (100), and (b) stacking of layers along the a crystallographic axis in the structure of compound 2b through hydrogen bond, C-H···π type and carbonyl interactions — S3

Figure S3: Layers of complexes arranged parallel to the crystallographic plane (001) in the structure of compound 3a through hydrogen bond interactions — S4

Figure S4 Layers of complexes arranged parallel to the crystallographic plane (010) in the structure of compound 3b — S5

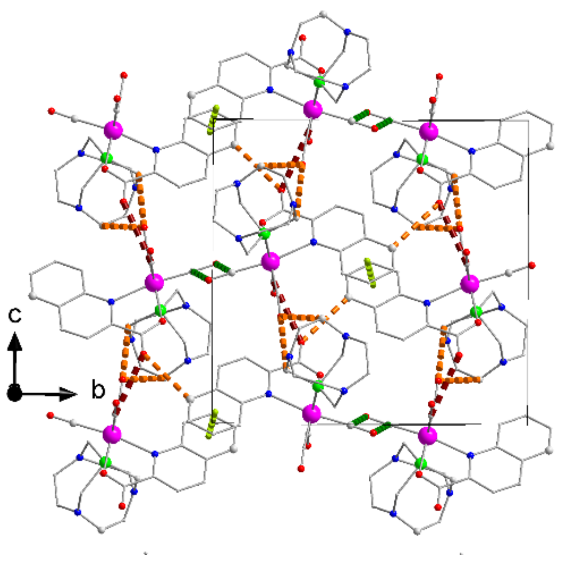

(a)

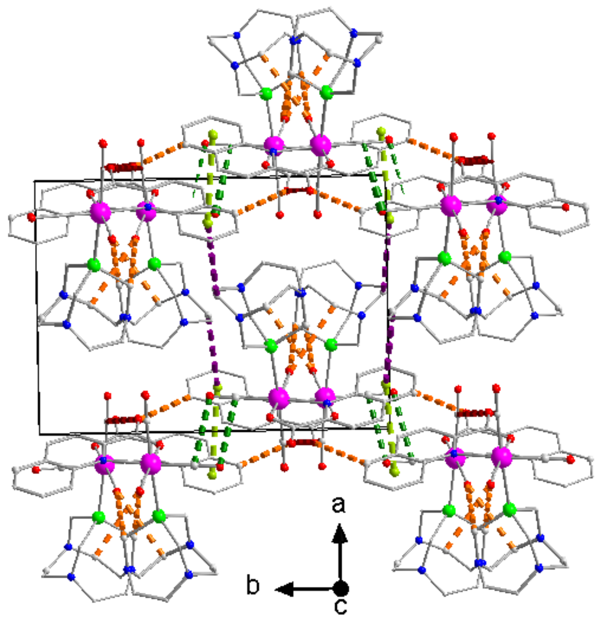

(b)

Figure S1. Intermolecular interactions in the structure of compound **1b** forming **a)** layers of complexes parallel to the crystallographic plane (100) and **(b)** Stacks of layers along the a crystallographic axis. Dashed thick dark green (antiparallel C3-O3···C3-O3) and dark red lines indicate carbonyl-carbonyl interactions (C1-O1···C-O5). Dark violet dashed lines indicate C19-H19···π interactions. Light green dashed lines indicate π···π

between quinaldic ligands and orange dashed lines indicate C18-H18A···O2, C14-H14B···O2 and C9-H9···O5 hydrogen bonds.

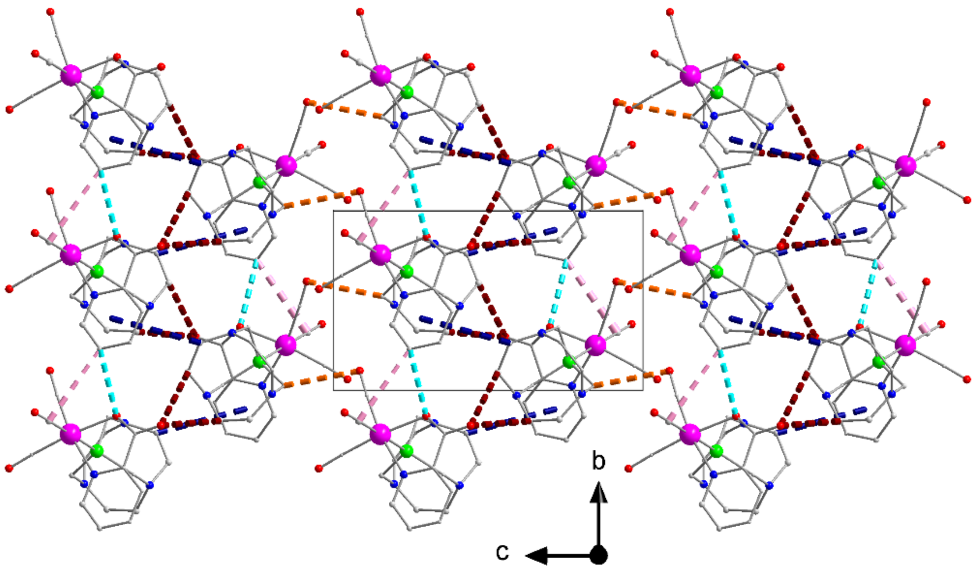

(a)

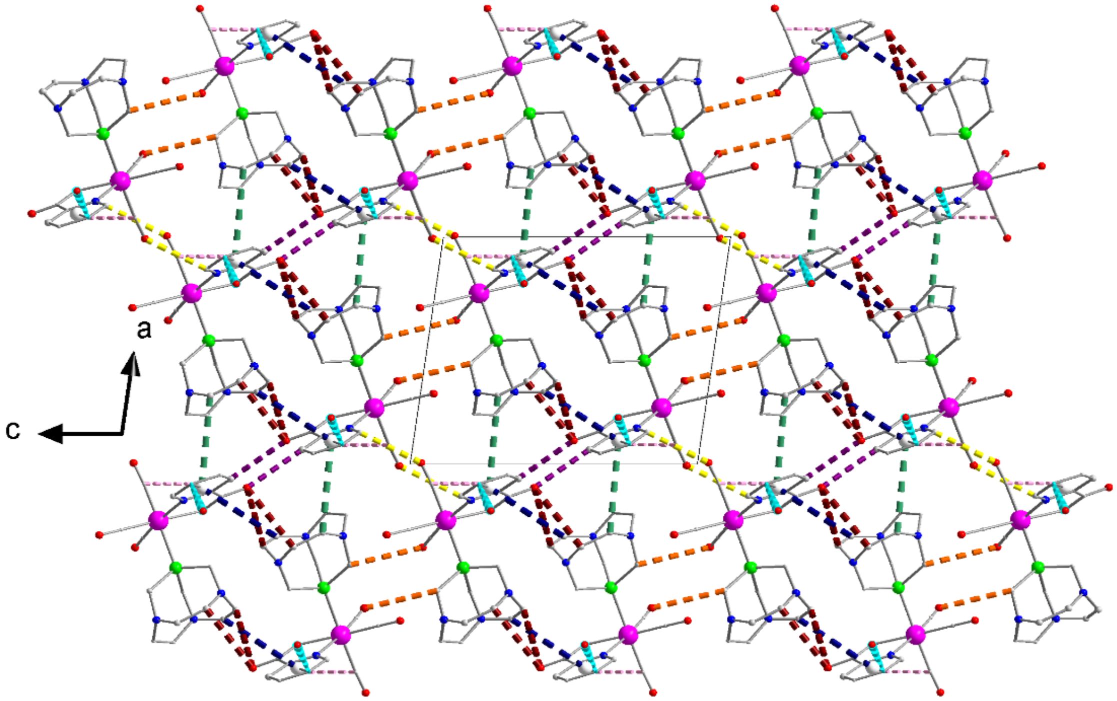

(b)

Figure S2. (a) Intermolecular interactions among neighbouring clusters forming layers of complexes arranged parallel to the crystallographic plane (100), and (**b)** stacking of layers along the a crystallographic axis in the structure of compound **2b** through hydrogen bond, C-H···π type and carbonyl interactions. The different type of hydrogen bond interactions which are indicated with dashed thick orange (C12-H12···O2), dark red (C14-H14A···O5 and C15-H15B···O5 pairs of interactions) and cyan (C8-H8···O4) dashed lines and C13-H13···Cg2 (C-H···π type of interactions) contribute in the formation of layers. The pink dashed lines indicate carbonyl C8-H8···C1 type of interactions, developed among clusters lying on the layers. Neighbouring layers interact through C6-H6···O5, C9-H9··O1 and C16-H16···Cg2 (C-H···π type of interactions) and are indicated with violet, yellow and dark green dashed lines respectively.

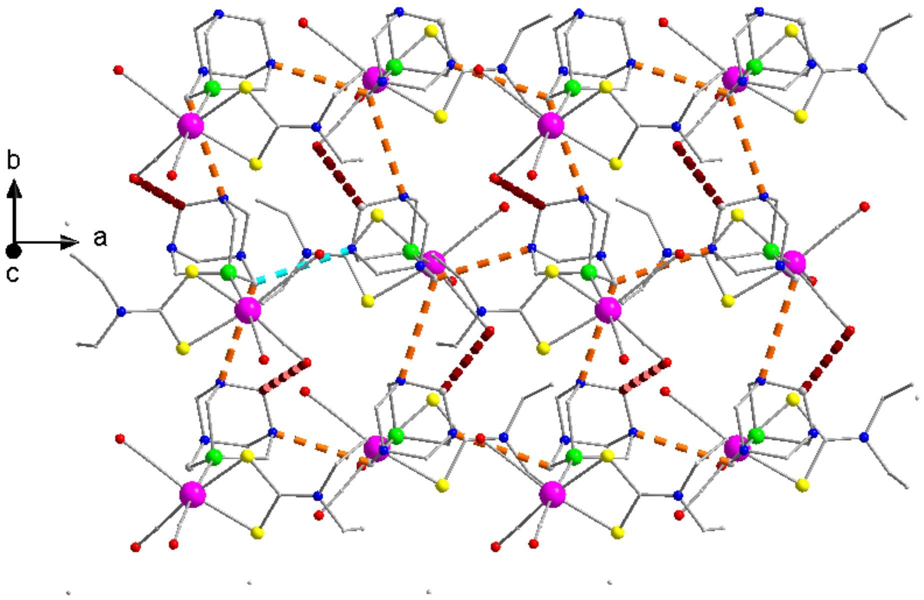

Figure S3. Layers of complexes arranged parallel to the crystallographic plane (001) in the structure of compound **3a** through hydrogen bond interactions. The dashed thick orange lines indicate C9-H9A···N4 and C9-H9B···N3 and the dark red one indicate C13-H13A···O1 hydrogen bonds.

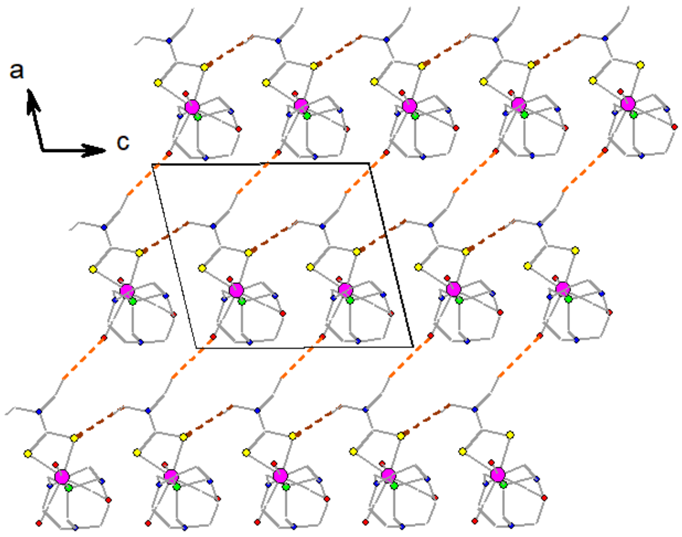

Figure S4 Layers of complexes arranged parallel to the crystallographic plane (010) in the structure of compound **3b** The dashed thick orange and dark red lines indicate C8-H8A···O2 and C5-H5···S2 hydrogen bonds respectively.
